# Experiences and outcomes of group volunteer befriending with patients with severe mental illness: an exploratory mixed-methods study in Colombia

**DOI:** 10.1186/s12888-021-03232-z

**Published:** 2021-05-06

**Authors:** Felipe Botero-Rodríguez, María Camila Hernandez, José Miguel Uribe-Restrepo, Camilo Cabariqe, Catherine Fung, Stefan Priebe, Carlos Gómez-Restrepo

**Affiliations:** 1 Department of Clinical Epidemiology and Biostatistics, Pontificia Universidad Javeriana, Bogotá, Colombia; 2 Department of Psychiatry and Mental Health, Pontificia Universidad Javeriana, Bogotá, Colombia; 3 Unit for Social and Community Psychiatry (WHO Collaborating Centre for Mental Health Service Development), Queen Mary, University of London, London, UK; 4 Hospital Universitario San Ignacio, Bogotá, Colombia

**Keywords:** Global mental health, Severe mental illness, Community mental health, Befriending, Volunteers

## Abstract

**Background:**

Improving care for patients with severe mental illness in Latin America requires effective strategies that are low-cost. One such strategy is a volunteering scheme, referred to as befriending, which seeks to support the social integration of patients. Despite positive reports in other world regions, this intervention has not been studied in Latin America. Whilst befriending programmes commonly form patient-volunteer dyads, group arrangements may be an alternative with some benefits. Here, we aim to explore the feasibility, experiences and outcomes of a group volunteer befriending intervention for patients with severe mental illness in Colombia.

**Methods:**

In this exploratory non-controlled study, 10 groups of five individuals were formed, each consisting of three individuals with schizophrenia or bipolar disorder and two volunteers from the community in Bogotá, Colombia. Each group was encouraged to participate together in social activities within their community over a 6-month period. Patients’ quality of life, objective social outcomes, symptom levels and internalised stigma were assessed before and after the intervention. Patients’ and volunteers’ experiences were explored in semi-structured interviews which were analysed using inductive content analysis.

**Results:**

Outcomes were available for 23 patients. Whilst their objective social situation had significantly improved at the end of the intervention, other outcomes did not show statistically significant differences. The interviews with participants revealed positive experiences which fell into five categories: 1) stigma reduction; 2) personal growth; 3) formation of relationships; 4) continuity and sustainability of befriending; 5) acceptability and feasibility of befriending.

**Conclusions:**

A volunteer befriending programme in small groups of two volunteers and three patients is feasible and associated with positive experiences of participants. Such programmes may also improve the objective social situation of patients. This low-cost intervention may be useful for patients with severe mental illnesses in Latin America.

**Trial registration:**

ISRCTN72241383 (Date of Registration: 04/03/2019, retrospectively registered).

## Background

Globally, severe mental illness is an important cause of morbidity and mortality. In Latin America, severe mental illness causes a third of the total years lost due to disability (YLD) and a fifth of the total disability-adjusted life years (DALYs) [1]. Low and middle-income countries (LMICs) face particular challenges in managing the burden of severe mental illness, including the lack of specialised professionals in mental health and poor health service coverage in large parts of countries [2–4]. These problems increase the treatment gap and the disease burden on patients, families, and communities [5]. Considering the impact of severe mental illness on individuals, communities and economies, mental health is increasingly regarded a global priority [6] and new community-based strategies for people with severe mental illness are required [7, 8].

One such strategy is support through befriending through volunteers, who are willing to spend some of their free time with people with mental illness without being paid or receiving other material benefits. Commonly, the volunteers do not have a mental disorder themselves which distinguished volunteer befriending from peer support. Befriending programmes vary depending on the aims, the characteristics of target group and participating volunteers and the local context. Evidence suggests that befriending programmes can reduce depression and loneliness and improve quality of life [9, 10]. A review of qualitative research on befriending shows that befrienders are usually motivated by both the interest to gain new helpful experiences and the will to help others in the community. Patients as well as volunteer befrienders report largely positive experiences [11].

A recent trial in the United Kingdom showed that befriending can help people with severe mental illness to overcome social isolation and establish more social contacts [12]. Social isolation is frequent among people with severe mental illness and has been associated with poorer quality of life, higher symptom levels, and higher levels of internalised stigma [13–15].

Against this background, we aimed to study the potential of volunteer befriending in Colombia as a LMIC. Following discussions with local stakeholders, the original idea of having the usual one-to-one model of befriending was modified. It was decided to form small groups of two volunteers and three patients. There were several reasons for this modification: A) With a group arrangement the meetings would still happen even if one or two participants would not attend. This was thought to be particularly important as regular attendance was difficult in a previous trial in London. B) Activities in small groups were seen as culturally more appropriate as they allow for a wider range of activities (e.g. in making music together). C) Meetings in groups should enrich the options for the sharing of experiences and mutual learning. D) Being in regular contact with more people was supposed to increase the opportunities for establishing relationships and tapping into informal networks. E) Pairing with another volunteer was intended to reduce the risk that volunteers feel overwhelmed and helpless.

A befriending programme in small groups was regarded as appropriate for the cultural context and is an intervention that – to our knowledge – has not been systematically evaluated in research before. We tested the feasibility of such a programme for patients with bipolar disorder and schizophrenia and explored the experiences and outcomes of participants.

## Methods

### Study design and participants

Between March and October of 2019, we conducted an exploratory non-controlled study in Bogotá, Colombia involving volunteers (recruited through flyers and word-of-mouth in the community) and patients with severe mental illness (recruited from mental health services). Full details are found in the study’s published protocol [16].

Eligible patients were aged between 18 and 65 years, were considered as having a severe mental illness by their psychiatrists with diagnoses of schizophrenia (CIE-10 F20) or bipolar disorder (CIE-10 F31), had capacity to provide written informed consent, and were willing to participate in activities with others that they did not know yet. Patients were excluded if they had a substance use disorder, organic psychosis or dementia, or were hospitalised at the time of recruitment.

Eligible volunteers had no history of mental disorders, were committed to supporting patients with severe mental illness, available to attend training, and would allow supervision throughout the intervention period.

### Intervention

Participants who met the selection criteria were matched to groups according to their common interests and the proximity of their homes [16]. All volunteers received training which covered information about the programme, the symptoms that the patients may present with, their responsibilities, resources for supervision, support from the research team, and emergency procedures. The training was delivered in a 4 hour session by the first author (FBR), and all volunteers also received a written manual with key information about the programme and its intended delivery (available from the authors). The volunteers were then instructed to contact the patients in their group and organise group meetings (lasting about 2 h) approximately every 15 days over the course of 6 months, for a total of 12 meetings per group. Within the first 2 months, volunteers were given the opportunity to move to a different group if they felt the original allocation was difficult for them.

Volunteers were encouraged to organise different indoor and outdoor activities in their group meetings (e.g., share a coffee, picnic in the park, and go for walks). Following each meeting, volunteers informed the research team - via text message or phone call – about the type of activity, the duration, and the content of discussions. Additionally, the research team organised activities, such as music activities, arts and crafts, and bird watching, which any group could attend together.

Patients and volunteers were given the opportunity to contact the research team at any time, discuss difficulties in the group and doubts about the programme, and request further supervision. For each meeting, a reimbursement of $20.000 COP ($5.5 USD) was given to each participant in order to cover transportation cost and support the activities.

Patients continued to receive treatment as usual, which for the sample in this study included a meeting with a psychiatrist about every 3 months and usually medication.

### Assessments

For volunteers, we collected socio-demographic data, volunteering history, and motivation for participating in the study with an open question.

For each patient, we collected socio-demographic information at baseline. Detailed information about the clinical history of patients was not available.

Outcomes were completed at baseline and at the end of the intervention after 6 months. Subjective quality of life was assessed using the Manchester Short Assessment of Quality of Life (MANSA), and taken as the mean score of 12 satisfaction items, each rated between 1 (could not be worse) and 7 (could not be better) [17]. The objective social situation was measured using the Objective Social Outcomes Index (SIX) [18]. This is a 4-item instrument which assesses employment, housing situation, social contacts and partnership using a scale from 0 to 6, with 6 representing the most favourable score. Symptom levels were assessed on the 24-item Brief Psychiatric Rating Scale (BPRS) [19]. Internalised stigma was assessed on the 29-item Internalised Stigma of Mental Illness scale (ISMI) [20], where each item is scored on a scale of 1 (strongly disagree) to 4 (strongly agree).

We interviewed convenience samples of 12 patients and 12 volunteers about their experiences. Interviews were conducted by a member of the research team (FBR) and recorded.

### Quantitative analysis

Baseline characteristics of volunteers and patients were analysed using descriptive statistics.

For assessing changes in outcomes MANSA, SIX, BPRS and ISMI, the mean differences between the baseline and 6-month measurements were analysed using Student’s paired t-tests for dependent variables. All analyses were conducted using R Project for Statistical Computing version 3.6.1.

### Qualitative analysis

The open questions about the motivation of volunteers were analysed using inductive content analysis.

The interviews of patients and volunteers were transcribed verbatim by an external transcription company. These transcripts were then reviewed by the research team (FBR and MCH) in order to ensure their accuracy. Finally, the anonymised transcripts were analysed using NVivo version 12.

An inductive content analysis was carried out according to the process described by Elo and Kyngäs [21] in order to explore the experiences and opinions of the participants. Although the volunteers’ and patients’ experiences of the programme were analysed separately to account for the variation in the different roles and potentially different experiences, the same protocol was followed. Two investigators (FBR and MCH) completed line-by-line open coding of the transcripts for all 24 interviews. These open codes were recorded on coding sheets, allowing initial categories to be formed. This grouping process was followed by further categorisation and abstraction in order to develop the sub-categories, generic categories and main categories, as part of an overall iterative process. The different raters in the research team had an agreement of > 80% on the inclusion and coding of data. For this paper, the original quotes in Spanish were translated into English by one member of the research team (MCH) and checked by another (SP) who speaks both languages.

## Results

A total of 30 patients and 20 volunteers provided written informed consent, completed the baseline assessments and participated in the study.

### Characteristics of participants

Table 1 summarises the characteristics of patients and volunteers.

**Table 1:** Characteristics of volunteers and patients

	Volunteers (*N* = 20)	Patients (*N* = 30)
**Sex**		
Female	13	18
Male	7	12
**Age, mean and SD**	24.5 (5.7)	41.2 (10.7)
**Marital Status**		
Single	20	21
Married	0	2
Free union	0	1
Separated	0	1
Divorced	0	4
Widow	0	1
**Ethnicity**		
Indigenous	1	1
Mestizo	7	11
White	7	8
Other	2	8
Does not know/No response	3	2
**Education level**		
Primary	0	1
Secondary	7	12
Technical	2	2
Professional	10	13
Masters	0	2
Does not know/No response	1	0
**Occupation (%)**		
Self-employed	7	12
Unemployed	4	12
Student	9	1
Homemaker	0	1
Retired due to disability	0	3
Retired due to age	0	1
**Housing Status**		
Lives alone	–	3
Shares with parents or family	–	24
Shares with friends or partner	–	3
**Primary diagnosis (%)**		
Schizophrenia	–	14
Bipolar disorder	–	16

*SD* Standard deviation

All patients were in some form of treatment as baseline and all but one were prescribed psychopharmacological medication (22 patients antipsychotics, 20 mood stabilizers, and 7 antidepressants).

Volunteers worked in different areas, such as sales, teaching, arts, and social management. Their weekly commitment to their jobs or education was 34.9 h (SD = 19.1). Their motivations for participating were mainly to serve their community, help others, grow as individuals, better understand mental health issues, reduce stigma, and raise awareness of mental illnesses.

At 6 months, we collected data from 23 patients. On average, patients attended 5.5 group meetings (range between 1 and 11) during the six-month intervention period. On average, group meetings were attended by 3.8 people.

### Outcomes

Table 2 shows the means for the assessment measures at baseline and at 6-months. During the intervention the SIX score increased, showing improvement in objective social situation. The difference was statistically significant. There was no significant change on the MANSA, BPRS and ISMI.

**Table 2:** Outcome measures at baseline and after the six-month intervention 6 months (*N* = 23; means and SDs)

	Baseline	6 months	95% CI	*p* value *(t test)*
MANSA	5.09 (0.96)	5.18 (1.03)	−0.56 - 0.38	0.695
SIX	4.17 (1.03)	4.61 (1.03)	−0.77 - -0.09	0.014
ISMI	1.99 (0.61)	1.92 (0.65)	−0.21 - 0.35	0.602
BPRS	1.26 (0.17)	1.27 (0.37)	−0.24 - 0.11	0.481

*CI* Confidence interval of difference. *MANSA* Manchester Short Assessment of Quality of Life. *SIX* Objective Social Outcomes Index. *BPRS* Brief Psychiatric Rating Scale. *ISMI* Internalized Stigma of Mental Illness

Considering the statistically significant difference on SIX, we conducted a post-hoc analysis to identify in which of the assessed areas (employment, accommodation and friendships/partnership) patients improved. Improvements were seen in the items on friendships (mean = 1.39; SD = 0.5 to mean = 1.65; SD = 0.49; *p* = 0.05) and as a statistical trend on employment (mean = 2.78; SD = 3.07 to mean = 3.45; SD = 3.08; *p* = 0.10), whilst there was no significant difference on the items assessing accommodation and partnership.

### Qualitative results

We interviewed 12 patients and 12 volunteers. Five overarching categories represent the general experiences of the participants. These were: stigma reduction; personal growth; formation of relationships; continuity and sustainability of befriending; and acceptability of befriending.

### Stigma reduction

Several volunteers expressed initial concerns that people with mental illnesses were dangerous or unstable and described feelings of fear before meeting the patients in their groups. Some of these concerns were based on how mental illness was portrayed in the media or on comments from relatives who advised them to be careful when participating in a study with psychiatric patients. However, some volunteers kept an open mind and described how they preferred to approach the patients with impartiality in order to genuinely understand them.

*“Before, I thought that one should be very cautious, when talking to them.” (volunteer 019, attended 7 meetings)*

*“Well, the expectations that I had were like to integrate myself with that part of society that has always been rejected but in which I have always really felt interested for that same reason that they were a population a little bit excluded … ” (volunteer 015, attended 8 meetings)*

*“ … at first I tried not to have expectations. I liked to think that I have no prejudices, but in the end one has them … ” (volunteer 026, attended 5 meetings)*

However, after the study, some of the volunteers’ perspective changed and they began to empathise with the patients in their groups, recognising that they shared similar experiences, interests, and habits. Consequently, this reduced the stigma attached to mental illness perceived by the volunteers.

*“ … they were patients and they were alone, … they only had their family, they were isolated and lonely, so I did feel an empathy with that, because I have also felt lonely and marginalised at some point.” (volunteer 014, attended 8 meetings)*

*“We realise that the truth is that they are ordinary people who have a disease like any other, which complicated certain things in life. But … they are more normal, it is ironic, I should not say it, but yes, they are more normal than one thinks they are.” (volunteer 026, attended 5 meetings)*

In contrast, other volunteers continued to view patients’ behaviours and appearances as shameful or embarrassing.

*“ … well, because we were walking down the street or something, and he acted strange, or sometimes he was very … the volume of his voice was sometimes very high, which, well, drew attention from other people and I sometimes felt that people pointed at me.” (volunteer 018, attended 9 meetings)*

*“ … he was very badly dressed, like, I don’t know, he looked in very bad condition, like seriously at first we began to doubt if he was our befriendee or if he was someone who lives in the street, that’s how badly he was dressed.” (volunteer 022, attended 2 meetings)*

Patients also reported that the befriending programme helped them to reduce their internalised stigma and feel more included and accepted by the community. Some patients stated that throughout the meetings, their background as a patient did not seem relevant for their interactions with the volunteers and that gradually the exchanges became less “hierarchical”, and more like conversations between “equals”.

*“ … we were all like super empathetic, like all good people, the illness was not noticeable, it was something great because we were like talking to any friend … ” (patient 021, attended 4 meetings)*

*“ … so I would talk about that issue, and look I think that many years will pass before we overcome this … [referring to mental illnesses]” (patient 009, attended 1 meeting)*

### Personal growth

Throughout the study, both patients and volunteers reported a positive impact on some personal aspects of their lives, ways of interacting with others, and self-care habits. As a result of seeing how the patients had to be mindful of the effect of their mental illness on their wellbeing, the volunteers also began to consider the necessity of caring for their own emotional needs and stress factors. Some of them reported daily problems in their emotional, professional, and academic lives.

“ … well, I would like to greatly improve the attention I give to things, I feel that sometimes I am very absent minded.” (volunteer 018*, attended 9 meetings*)

“ … this could happen to anyone who is under very high levels of stress, or in very complicated situations, so, first be aware that you have to take care of yourself … ” (volunteer 026*, attended 5 meetings*)

Some patients also spoke about an improvement in their quality of life as they became more sociable and felt that their symptom levels were reduced. They mentioned that they were initially nervous about approaching new individuals, but through participating in different activities and becoming more comfortable in conversations with group members, they realised that they could actually have fun in these situations. Some of the patients even reported to feel more in control of their personal lives due to the interactions with the volunteers.

*“ … I liked it a lot, it made me more open to other individuals, not only the small social circle that I have, yes, I liked it a lot.” (patient 021, attended 4 meetings)*

*“It generated a lot of impact because I learned to know more, I learned to orientate myself, to walk, to breathe, to think, to reflect, meditate, to share with other people, it helped me a lot, it was a positive development.” (patient 023, attended 7 meetings)*

Some volunteers however did not report a benefit and felt rather overwhelmed by the responsibility to coordinate the group. This led them to attend only a few meetings and prevented them from achieving a rewarding experience.

*“ … I didn’t see any solution, no, I seriously had to abandon this and have someone else take this, that maybe could organise the schedules better, or something like that, then you told me no that it was going to end and so I stayed.” (volunteer 009, attended 2 meetings)*

### Formation of relationships

New, special bonds were formed between the participants. The trust generated within these ties led them to have honest and open conversations about their personal and emotional lives.

*“Yes, some told me about their love relationships, they also fall in love. I also told them about my love relationship, it was like a fairly reciprocal relationship, to the extent that we opened our hearts and mentioned private things … ” (volunteer 014, attended 8 meetings)*

*“One felt trust, they inspired trust.” (patient 023, attended 7 meetings)*

Additionally, the volunteers highlighted that the patients were, in return, always available to listen to them and offer them advice when necessary.

*“ … for me it was a very nice experience, like going there to vent and to share.” (volunteer 026, attended 5 meetings)*

*“ … one time I said I was very overwhelmed and one of them gave me some very good advice … ” (volunteer 027, attended 5 meetings)*

Furthermore, the distinction between those participants with and without a mental illness became blurred over the course of the 6-month programme. Although volunteers were the ones who handled the money for the expenses and initially arranged the meetings, volunteers and patients described that their relationships evolved through solidarity and an environment free of judgment and criticism, where each individual participated in the organisation of later events.

*“ … realising that, let’s say, that although people have a diagnosis or not, we were all five people, for me everything was always normal, five people sharing, chatting, talking about life, movies, things and that.” (volunteer 027, attended 5 meetings)*

*“I felt very good because they are like relaxed people, like very friendly, they weren’t arrogant people, nor did they judge us, or anything.” (patient 030, attended 7 meetings)*

Other participants however who saw each other only a few times engaged in superficial conversation and formed friendly, but not close, relationships.

*“No, honestly no, we never got to talk about deep topics or specific topics about one another, like to give out information that, that I wouldn’t give another person. I don’t know I consider myself an open person, but, well, something like a confidante or something like that, no.” (volunteer 028, attended 2 meetings)*

### Continuity and sustainability of befriending

The participants also described barriers to arranging meetings during the 6-month period, and to continue with the meetings after the end of the programme. In particular, the volunteers regretted that after the 6 months, there was no formal closure to the befriending relationship, despite being encouraged at the beginning of the study to continue the meetings without financial support from the research team. Additionally, the busy schedules of the volunteers made organising further meetings difficult.

*“We never completed a closing, we just tried to arrange several meetings, that is, before the six months were up. We suggested five or six dates, but then it did not fit into one person’s schedule nor another, then the other person did not answer, as like we eventually stopped trying and then we lost contact. In other words, there was no closure as such...” (volunteer 022, attended 2 meetings)*

*“Look, we haven’t spoken again, but I think it’s because of the academic load that I’ve honestly had...” (volunteer 015, attended 8 meetings)*

Some patients wanted to continue and also encountered difficulties in co-ordinating the different schedules of everyone in the group.

*“I would like the opportunity to meet again, but it did not happen. I understand them, they organise the conversation and it is difficult that all agree.” (patient 019, attended 2 meetings)*

*“ … for volunteering to continue, like it has, if anyone else wants to come along, … .they are welcomed.” (patient 023, attended 7 meetings)*

### Acceptability and feasibility of befriending

At the end of the study, several participants expressed appreciation for their encounters and felt that the programme was very beneficial to the patients.

*“I believe that this type of experience is like a new treatment for them, which helps us to realise that they may have their problems, but it is not as serious as you imagine, it is not something from another world.” (volunteer 028, attended 2 meetings)*

*“It is a good thing because they are trying to help us people with mental disorders, not to be in the same routine every day at home, and go out. Or that maybe the family circle isn’t the only one that exists, instead that one can also have friends.” (patient 030, attended 7 meetings)*

Participants also spoke about the many activities that the city offered them, such as visiting the park, going to the library, and bird watching. This variety facilitated the feasibility of the meetings, and allowed the participants to have enjoy activities throughout the study and appreciate the cultural spaces in the city.

*“We also completed an activity … of riding little boats. It was super cool. I had never done that in my life and neither had they, that moment was very exciting … ” (volunteer 015, attended 8 meetings)*

*“ … within the cultural agenda of Bogota there are many things to do.” (patient 009, attended 1 meeting)*

## Discussion

This is the first study to evaluate a volunteer befriending programme in Colombia and – to our knowledge – the first one assessing a group befriending programme in the world. In the group programme patients with schizophrenia and bipolar disorders were linked with volunteers who – with an average of 24.5 years – were much younger than the patients, and were mostly self-employed or students with full time commitments. The findings show that the six-month intervention is feasible. All planned groups materialised, the meetings were attended well, and experiences of participants were largely positive. Participating in the programme was associated with significant improvements of the objective social situation, whilst we did not find significant changes on scales assessing subjective quality of life, symptom levels and self-stigma. However, it has to be taken into account that patients at baseline already had relatively favourable scores on all scales, i.e. with high mean scores in MANSA and SIX and low levels of symptoms and self-stigma, as compared with other patient groups with severe mental illness reported in the literature [12, 17, 18, 20].

The study has several strengths. Adherence rates were much better than in a trial on volunteer befriending in the United Kingdom [12], and as a mixed-methods study it combined quantitative and qualitative evaluation methods. The quantitative assessment used established scales and well-trained researchers. The qualitative evaluation followed a rigorous process of data coding and analysis.

The study also has limitations. The sample size was rather small and the loss of patients to follow-up reduced the sample size for the analysis even further. The absence of a control group made it impossible to establish effectiveness. The very favourable baseline scores with low symptom levels, low self-stigma and positive subjective quality of life made it difficult to find further improvements during the intervention. They may also limit the extent to which the findings can be generalised to more symptomatic patient groups with higher self-stigma and poorer quality of life. The qualitative data suggest positive effects on symptoms and self-stigma which is not reflected in the quantitative ratings, again potentially because of a floor effect in the respective scale scores.

The great variability of volunteer befriending programmes raises the question as to what extent they are comparable and represent one coherent type of intervention. Whilst our group approach had not been evaluated in systematic research before, the type of volunteers recruited in this study are different from those dominating in the literature on volunteer befriending in mental health. As in most studies, the majority of volunteers were female [11, 22]. However, most of our volunteers were neither retired nor above 50 years of age as has been found in most other programmes [11, 22]. The volunteers in our study had full time commitments as self-employed professionals or students and all lived alone. Importantly, they were much younger than the patients which must have had an impact on the type of relationship they established with the patients. The characteristics of the volunteers are similar to those in a recent randomised controlled trial in London [12], where most of the volunteers were young students. Like our study, the trial in London found a significant improvement only on measures of the objective social situation – albeit not to the same extent as in our study – and not on other outcomes. One can only speculate as to whether the large improvement in the social situation has been facilitated by the group approach. The improvement reflects that patients had established more friendships and also, as a trend, that they had found employment. Forming groups – rather than one-to-one dyads – may come with more encouragement to actively go out and seek friends and work. It may also widen the learning from peers as well as from volunteers and facilitate the access to informal networks of the other group members which may have helped to find work and new friends. Informal networks may be larger in Colombia than in countries where previous research on befriending has been conducted [23].

In in-depth interviews, both patients and volunteers reported positive experiences. They highlighted how the intervention helped them to take advantage of the urban environment in their city, to achieve change in different respects and to learn from others. Most importantly, patients appreciated the different support they received from their groups.

In the interviews, the participants also made recommendations for how to improve the intervention. They suggest a stricter accountability for participants arranging meetings, increasing financial support, having the research team facilitate more joint activities, and increasing the group size. Participants also raised the role of the patients’ family within the programme. Several patients reported that they received support from their families to participate in the programme, and some patients even brought a family member to the activities. Family support has been associated with a greater perception of the need for personal care, which has been viewed as a predictor of health services use [24]. The close-knit family dynamics within Latin-American cultures might influence participation in the befriending programme [25]. Future research may explore this further and assess family dynamics in other cultures by integrating family members in befriending programmes.

Volunteers reported experiences similar to other studies on befriending, which showed a sense of accomplishment and satisfaction as volunteers were able to contribute to the community [26]. The motivations of the volunteers in our study included opportunities to: serve the community, help others, grow as a person, further understand mental health issues, diminish stigma, and raise awareness about mental health. Similar motivations were also found in a systematic review [11], in which individuals were grouped based on their aspiration to serve others and give back by getting involved in the community, testing their capacity as a volunteer, finding explanations for their own behaviours, testing their career aspirations, learning more about health services, and meeting new individuals. Previous literature reports that volunteering is associated with better physical and mental wellbeing, better perception of health and quality of life, and fewer depressive symptoms [27–30]. However, we did not assess these outcomes in volunteers, and they may be relevant to assess in future research.

Volunteer befriending programmes require some organisational support, but are still low cost for health services. They require limited input for the recruitment, training and potential supervision of volunteer befrienders, and some expenses for activities may be reimbursed. Beyond this, these programmes do not generate costs for the health care organisations. They are also independent of several barriers to accessing traditional medical services such as time, distance, and costs associated with travel to services or lack of health care coverage. All such barriers can be relevant in Colombia and other LMICs.

The study was conducted in Bogotá, which is a metropolis with a population of more than seven million people. Befriending programmes may have to be amended for rural areas where the social fabric and the barriers to traveling can be different.

## Conclusion

Despite the increase in insurance coverage in Colombia, access to health services has decreased significantly according to the data from the National Quality of Life Survey, negatively affecting the health outcomes of the nation’s population [5, 31] and limiting the care for people with severe mental illness. Our study reports positive experiences and outcomes of a group befriending programme within the Colombian context. As this intervention is also low cost to services and require only limited input from qualified health care professionals, it may represent a useful approach in LMICs such as Colombia, ideally complementing and not replacing conventional treatment. We believe our study justifies wider implementation of befriending programmes, further development of group programmes and more research to evaluate different models of befriending in the Latin American culture.

## Data Availability

The datasets used or analysed during the current study will be available from the corresponding author on reasonable request.

## Abbreviations

MANSA: Manchester Short Assessment of Quality of Life
SIX: Objective Social Outcomes Index
ISMI: Internalized Stigma of Mental Illness
BPRS: Brief Psychiatric Rating Scale
CSRI: Client Service Receipt Inventory
